# Posterior uveal biopsy and the trans-scleral Essen forceps biopsy technique

**DOI:** 10.1038/s41433-024-03394-6

**Published:** 2024-10-14

**Authors:** Vanessa Otti, Caroline Thaung, Hardeep Singh Mudhar, Bertil Damato, Mandeep S. Sagoo, Hibba Quhill

**Affiliations:** 1 University College London Medical School, London, UK; 2 Ocular Oncology Service, Moorfields Eye Hospital, London, UK; 3 Department of Histopathology, Royal Hallamshire Hospital, Sheffield, UK; 4 NIHR Biomedical Research Centre for Ophthalmology at Moorfields Eye Hospital and University College London Institute of Ophthalmology, London, UK; 5 Ocular Oncology Service, Royal Hallamshire Hospital, Sheffield, UK

**Keywords:** Eye cancer, Cell biology

## Abstract

**Background:**

Differentiating neoplastic and non-neoplastic uveal tumours can present a diagnostic challenge; intra-ocular biopsy may be necessary. The novel trans-scleral Essen Forceps biopsy (TSEB) technique can improve diagnostic yield compared to fine needle aspiration biopsy (FNAB). We present a case demonstrating the technique and its added value. We also review the success rate of TSEB performed at two tertiary eye centres.

**Methods:**

Retrospective case report and consecutive case series from August 2021 to March 2023. Inclusion criteria were patients who underwent TSEB of posterior uveal lesions from Moorfields Eye Hospital and Sheffield Teaching Hospitals in the United Kingdom. The outcomes were biopsy success rate and complication rate

**Results:**

Eleven biopsies met the inclusion criteria. Eight (73%) were successful, which comprised six uveal melanomas, one melanocytoma and one extranodal marginal zone (ENMZ) lymphoma. One TSEB did not yield tissue for histological examination because of perioperative sample handling. Two (18%) biopsies were histologically inconclusive; both were treated as uveal melanoma on clinical grounds or repeat biopsy. The only complication was vitreous loss and retinal hole without retinal detachment in one eye with a very posterior, shallow choroidal lesion.

**Conclusion:**

TSEB is an effective alternative to established biopsy techniques, yielding larger tissue samples than FNAB with intact tissue architecture. We recommend adding TSEB to the armamentarium of the ocular oncologist.

## Introduction

Diagnosis of uveal melanoma (UM) is commonly based on clinical findings and multimodal imaging in the vast majority of cases [1]. However, some intraocular masses pose a diagnostic challenge on clinical examination and imaging alone, and a tissue diagnosis is desirable. Some conditions can masquerade as UM; such as naevi, posterior scleritis and giant pigment epithelial detachment [2–7]. Though most UM patients present with painless visual disturbance or are asymptomatic at presentation, UM can rarely present atypically, mimicking other conditions, such as uveitis, scleritis, sarcoid or tuberculous granulomas [8, 9]. Differentiating these conditions can prove a diagnostic dilemma and intraocular diagnostic biopsy may be necessary [10].

Fine needle aspiration biopsy (FNAB) is the established technique for taking a tumour sample. However, its diagnostic accuracy can be limited by the location and access to the lesion, small yields and the requirement for specialist interpretation by an ophthalmic histopathologist or cytopathologist. The FNAB sampling method disrupts tissue architecture, making interpretation challenging in cases [1, 11–13]. The Essen forceps biopsy, as described by Akgul et al. [14], is a suitable alternative to FNAB [11]. The Essen forceps were designed for transvitreal approach biopsies. Herein, we describe our experience of using the Essen forceps to take tumour biopsies *ab externo*, utilising a partial-thickness scleral flap technique. This study describes the technique and reviews the success and complication rates of trans-scleral Essen biopsy (TSEB) technique performed at two tertiary ocular oncology centres in the UK. We also report an illustrative case highlighting the added value of TSEB in selected cases.

## Methods

We retrospectively reviewed medical and histopathological records of patients undergoing uveal TSEB at two national ocular oncology centres: Moorfields Eye Hospital, London, and Royal Hallamshire Hospital, Sheffield. The biopsies took place between August 2021 and March 2023. Approval was obtained from the Audit Department of Moorfields Eye Hospital (1331). The study adhered to the tenets of the Declaration of Helsinki.

Data collected included patient demographics, lesion location and size, success rate (measured as a biopsy that yielded a tissue diagnosis), biopsy result, complication rate and, in cases of biopsy failure, reasons for failure.

### Trans-scleral Essen biopsy technique

**Fig. 1:**
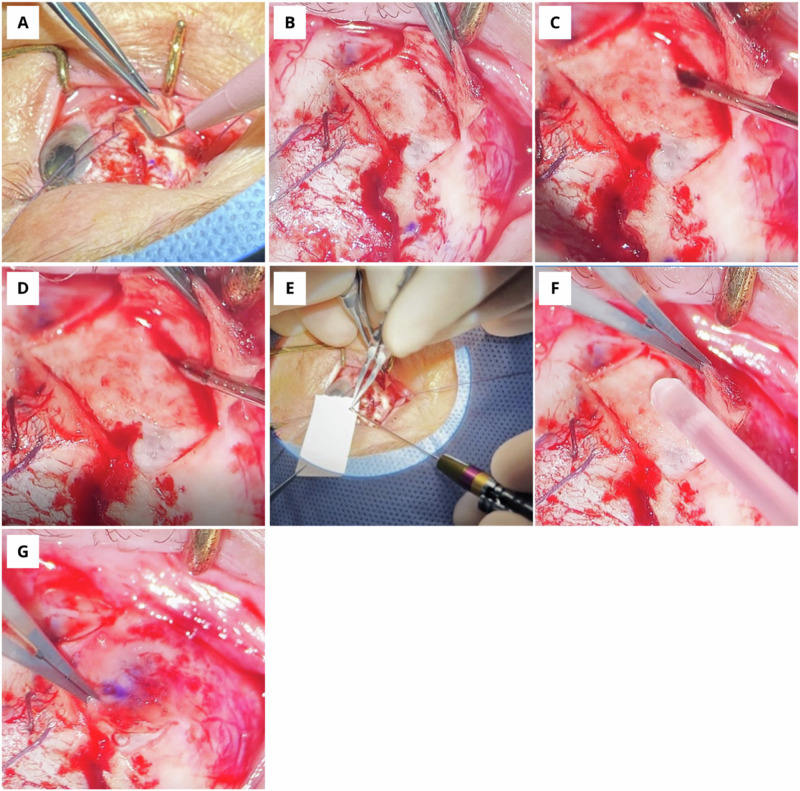
Trans-scleral tumour biopsy with Essen forceps. Following scleral exposure and marking of the tumour site, a fornix based partial thickness scleral flap is created (**A**) with the hinge over the thickest part of the tumour. Near the hinge of the flap, a 2-3 mm full thickness sclerostomy is created with a blade (**B**) giving access to the intraocular structures. The Essen forceps is passed into the lesion to be biopsied with the jaws open (**C**). The forceps are closed and the tissue sample remains within the jaws of the forceps (**D**). The tissue is placed on sterile card under the microscope to ensure the samples are safely retrieved (**E**). This process is repeated as often as necessary, sampling different parts of the lesion of interest each time. NB: for very thin lesions, the forceps should be passed along the inside of the sclera, parallel to the plane to avoid perforating retina. To minimise the chance of tumour cell seeding, the flap is closed by applying a small drop of cyanoacrylate glue (**F**). The flap is rapidly closed (**G**) with gentle pressure over the sclerostomy using a cotton bud. If needed, further glue can be applied to ensure full closure or 9-0 non-absorbable sutures can be pre-placed at the flap corners.

A partial-thickness scleral flap is created over the lesion to be biopsied. To gain access to the intraocular structures, a linear, 2 mm, deep-scleral incision is made close to the ‘hinge’ of the scleral flap. The Essen forceps is passed into the lesion with the jaws of the forceps open, tangentially so as to hug the inner scleral surface to prevent inadvertent retinal perforation. The jaws are closed, retaining the sample in the forceps. The sample is collected on a pre-soaked cellulose sponge or card. Multiple bites can be taken from different parts of the tumour by altering the direction of insertion of the forceps. The samples are inspected under the microscope to ensure retrieval. To minimise the chance of tumour cell seeding, bipolar cautery is applied to any visible cells. The flap is closed by applying a drop of cyanoacrylate glue and gentle pressure over the scleral flap using a cotton bud. This holds any potential tumour seeds at the biopsy site, in ‘suspended animation’, where they can be treated by subsequent radiotherapy if needed. Further glue can be applied to ensure complete closure, or alternatively, 9-0 non-absorbable sutures placed at the flap corners. Figure 1 illustrates the steps.

All biopsies obtained with this technique were fixed in standard 4% neutral buffered formalin and processed with standard pathology techniques to paraffin wax blocks. Paraffin sections were cut at 4 microns, stained with haematoxylin and eosin (H & E) and examined with a standard light microscope. Depending on the pathological findings, paraffin sections were exposed to appropriate immunohistochemistry and ancillary molecular genetic analysis.

## Results

### Case report

**Fig. 2:**
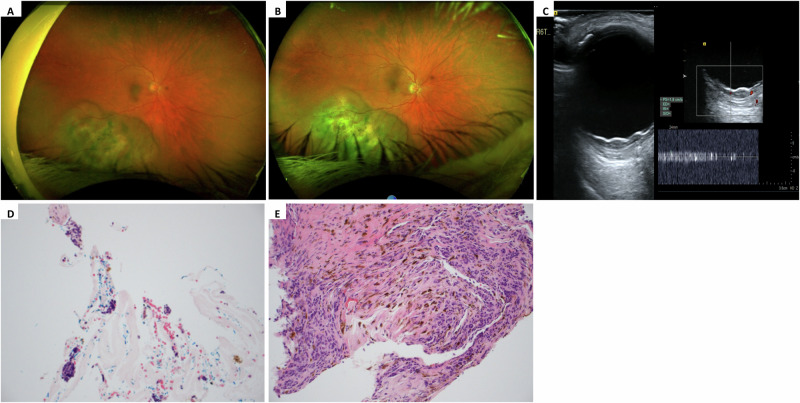
Multimodal clinical imaging of the case described. **A** Optos widefield fundal imaging at initial presentation with acute red eye. **B** Optos widefield fundal imaging three months after presentation following treatment with topical steroids. There was no regression in the choroidal lesion and subtle growth at the superior edge. **C** B-mode ultrasound of the choroidal mass at presentation, 2.1 mm (height) × 11.7 mm (longitudinal base) × 14.3 mm (transverse base), with low to medium echogenicity. There was a possible, slow internal blood flow as the recorded velocity in one vessel was 1.8 cm/s. **D** Histopathology of the specimen from the trans-vitreal biopsy of the right choroid which included tiny clumps of atypical cells. **E** Histopathology specimen obtained by Trans-scleral Essen Forceps biopsy (TSEB) of the right eye, stained with H & E at low magnification. This shows fibroconnective tissue with scattered melanin and an infiltrate of atypical cells with hyperchromatic nuclei, confirming malignant choroidal melanoma.

A 70-year-old white male presented with a 13-day history of red right eye associated with pain and blurred vision. He had no relevant past ocular or medical history. Best corrected visual acuity was 20/40 in the right eye and 20/20 in the left eye. Left eye examination was unremarkable. The right eye conjunctiva was both chemotic and injected over the inferotemporal quadrant. Intraocular pressures were normal. Dilated fundoscopy of the right eye showed an inferior shallow exudative retinal detachment, with an associated predominately amelanotic, irregularly shaped choroidal mass in the inferotemporal quadrant, without orange pigment, exudation or visible intralesional vessels (Fig. 2A).

Optical coherence tomography (OCT) through the area of the mass showed and irregular surface profile and trace subretinal fluid. B-scan ultrasound (US) confirmed a shallow choroidal mass, 2.1 mm in thickness, with a base of 11.7 mm × 14.3 mm, with low to medium echogenicity (Fig. 2C). There was minimal internal blood flow on Doppler US.

At this stage, the clinical picture suggested an inflammatory aetiology with associated choroidal mass. The differential diagnosis included nodular scleritis, choroidal metastasis from an unknown primary, and choroidal melanoma. The patient was commenced on a trial of topical dexamethasone eye drops and monitored closely.

Topical steroid treatment improved the symptoms of swelling and redness. However, dilated fundoscopy three months after presentation showed the choroidal lesion had not regressed (Fig. 2B). The patient was concurrently referred for systemic screening for a possible unknown primary malignancy, and underwent trans-vitreal biopsy of the choroidal mass using a 25 G vitreous cutter.

Systemic screening, including whole-body positron emission tomography-computed tomography (PET-CT), magnetic resonance imaging (MRI) head and neck and dermatology assessment, did not identify a systemic malignancy.

Histopathological evaluation of the trans-vitreal choroidal biopsy revealed a few small clusters of hyperchromatic atypical cells with scanty cytoplasm. Immunohistochemistry was negative for the melanocytic markers Human Melanoma Black 45 (HMB45) and melanoma antigen recognised by T cells 1/MART-1 (Melan-A). Immunohistochemistry for AE1/AE3, CD3 and CD79a were also negative, rendering carcinoma and lymphoma unlikely (Fig. 2D).

**Fig. 3:**
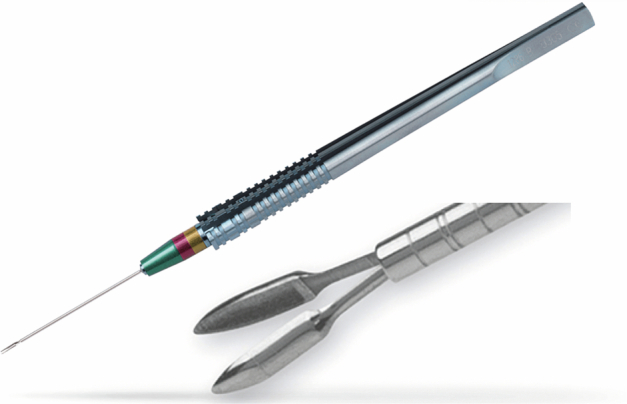
Essen Biopsy Forceps, with shaft engraved depth indicator. Developed by the Dutch Ophthalmic Research Centre and Department of Ophthalmology, University of Duisburg-Essen, Zuidland, The Netherlands [14]. Source: the Dutch Ophthalmic Research Center (D.O.R.C) Global Digital Marketing Manager, also available at [https://dorcglobal.com/product/akgul-biopsy-forceps-sharp-tip-23-gauge-06-mm]. License: Permission granted by D.O.R.C to use images for research purposes on 02/08/2022.

Given the ongoing diagnostic uncertainty, a second biopsy was undertaken. The TSEB technique was used (as described above, see Fig. 1), as initially described Damato et al. [15], utilising the Essen Forceps (23-gauge/0.6 mm) [14] (Fig. 3, Dutch Ophthalmic Research Center, Zuidland, The Netherlands), which was designed for trans-vitreal use [14–16].

Histopathological examination of the TSEB revealed several 1 mm fragments with intact tissue architecture. This consisted of fibroconnective tissue with scattered melanin, consistent with choroidal origin. An infiltrate of atypical cells with hyperchromatic nuclei was also identified (Fig. 2E). The atypical cells were immuniohistochemically positive for HMB45 and Melan-A and negative for cytokeratins, consistent with malignant melanoma. Subsequent genetic analysis of the tissue revealed loss of chromosome 3 and gain of chromosome 8: supporting a diagnosis of primary uveal melanoma with increased metastatic potential. Cytokeratin markers were all negative, as previously, ruling out metastatic carcinoma.

The patient subsequently underwent treatment of the choroidal melanoma with Ruthenium 106 brachytherapy at a dose of 100 Gray to the apex and continues under surveillance for metastasis as per local protocol.

### Review of TSEB performed at our centres

Data were extracted for 17 intraocular TSEB cases, of which 11 were of the posterior uvea and, therefore, eligible for inclusion. The details are summarised in Table 1.

**Table 1:** Patients undergoing trans-scleral Essen biopsies (TSEB).

Variable		*N* (%)
**Mean Age**	66 (range 50–88)	-
**Gender**	Male	10 (91)
	Female	1 (9)
**Laterality**	Right	8 (73)
	Left	3 (27)
**Lesion Location**	Temporal	3 (27.5)
	Inferotemporal	3 (27.5)
	Inferior	1 (9)
	Inferonasal	1 (9)
	Nasal	1 (9)
	Superonasal	1 (9)
	Superior	1 (9)
**Anterior or Posterior**	Anterior	8 (73)
	Posterior	3 (27)
**Uveal location**	Choroid	9 (82)
	Ciliary Body	2 (18)
**Mean Lesion Thickness (mm)**	4.9 (range 1.4–11.2)	-
**Mean Lesion Diameter (mm)**	13.9 (range 6.3–22.8)	-
**Biopsy result**	Choroidal/ciliary body melanoma	6 (54)
	Choroidal melanocytoma	1 (9)
	Inconclusive	2 (18)
	Contaminants only	1 (9)
	ENMZ Lymphoma	1 (9)
**Success rate**	Total	8 (73)
**Treatment**	Observation	1 (9)
	Plaque Brachytherapy	4 (36.5)
	External Beam Radiotherapy	1 (9)
	Enucleation	4 (36.5)
	Proton Beam	1 (9)
**Complications**	Vitreous Loss	1 (9)
	None	10 (91)

Demographics, clinical features, results and complications of patients undergoing TSEB between August 2021 and March 2023. Data were collected retrospectively from medical and histopathological records for 17 intraocular TSEB cases, of which 11 eligible for inclusion.

*ENMZ* extra-nodal marginal zone

In total, ten biopsies yielded informative samples (91%), with eight (73%) providing a tissue diagnosis and were therefore ‘diagnostically successful’. Two (18%) biopsies were histologically inconclusive despite yielding copious tissue; one was treated as choroidal melanoma based on clinical features, and the other underwent a subsequent trans-scleral incisional biopsy which confirmed choroidal melanoma.

One (9%) TSEB failed to yield any uveal tissue. The sample was inadvertently mishandled peri-operatively after the biopsy site had been sealed, while being transferred to the specimen pot. It is likely that the sample was lost in this process as no other TSEB failed to yield tissue. This patient was treated as a presumed metastasis from a known disseminated primary adenocarcinoma.

In our cohort, one (9%) complication occurred. This patient had a posterior diffuse choroidal tumour, located under the inferotemporal vascular arcade, with a maximum thickness of 2.6 mm. TSEB was performed because the vitreoretinal team locally was reluctant to biopsy transvitreally without using silicone oil due to the presence of multifocal serous retinal detachments. During TSEB, the retina was perforated with resulting low-volume vitreous loss. Fortunately, the patient had an uneventful post-operative recovery and did not develop retinal detachment, likely due to the very posterior location of the small retinal hole. The post-operative visual function was not affected by the biopsy, which confirmed choroidal extranodal marginal zone choroidal lymphoma. The failed TSEBs are detailed in Table 2.

**Table 2:** Inconclusive Trans-scleral Essen Forceps biopsies (TSEB).

Patient number	Lesion location	Lesion thickness (mm)	Max lesion diameter (mm)	Reason for biopsy failure	Repeat biopsy?	Ultimate Diagnosis	Treatment
1	Anterior, inferotemporal quadrant	2.8	6.3	Inconclusive. Bland melanocytic proliferation	No	Presumed ciliary body melanoma	Plaque brachytherapy
2	Anterior, superonasal quadrant	9.5	16.5	Inconclusive, melanomacrophages biopsied only	PLSU	Confirmed choroidal melanoma	Enucleation
3	Anterior, inferonasal quadrant	5.7	13.2	Sample dropped -Surface epithelial contamination and no uveal tissue received.	No	Presumed metastases from disseminated renal cell carcinoma.	Observation

Clinical features and results of patients undergoing TSEB between August 2021 and March 2023 who had an inconclusive initial biopsy. Data were collected retrospectively from medical and histopathological records. Three patients had inconclusive biopsies with one requiring repeat biopsy.

*Anterior* anterior to the equator of the globe

*PLSU* partial lamellar sclerouvectomy

## Discussion

It is uncommon to require a biopsy to diagnose an intraocular mass. Akgül developed the Essen Forceps for trans-vitreal biopsy of intraocular tumours. This instrument is a standard 23-gauge/0.6 mm intraocular forceps modified with inner grasping surfaces at the tip branches of the forceps (Fig. 3), making it possible to capture approximately 1 mm^3^ pieces of uncrushed tissue, maintaining tissue architecture [12, 15].

This study details the trans-scleral Essen Forceps biopsy (TSEB) technique in posterior uveal lesions. In the illustrative case report presented, the biopsy sample achieved was superior to that obtained trans-retinally using the 25 G vitrectomy cutter, completely changing the diagnosis and management of the patient. A second biopsy could have been attempted by FNAB or incisional partial lamellar sclerouvectomy (PLSU); however, the lesion’s posterior location and shallowness rendered access challenging and higher risk for retinal puncture.

For choroidal lesions, the diagnostic yield of FNAB typically ranges from 65–88% in the literature [1, 13]. With TSEB and incisional biopsies, larger samples can be obtained with preservation of tissue architecture [10]. As the surgeon visualises the uveal tissue during the procedure, there is less risk of non-diagnostic biopsies as the surgeon can complete further passes until tissue is retrieved, even in more solid lesions. Handling of the tissues is easier with Essen forceps than with the use of standard forceps, scissors or scalpel, and its blunt tip is less likely to perforate the overlying retina.

At our centres, trans-vitreal biopsy is a commonly employed technique for posterior uveal lesions due in part to difficulties with access trans-sclerally and can be performed using Essen forceps or a 25 G vitreous cutter. While the latter approach has a reported success rate of 93% [17], in our case report, histopathological and immunohistochemical analyses were non-diagnostic, most likely due to the limited amount of tissue sampled.

This study of TSEB presents the technique in detail and reviews the success and complication rates of this method. Overall, 91% (10 of 11) of TSEB yielded tissue, and is likely to have been higher had the one specimen not been mishandled during the procedure. Of those TSEBs which yielded uveal tissue, 80% (8 of 10) were of sufficient quantity and quality to allow for diagnosis of the uveal lesion. The two TSEBs with histologically inconclusive results showed either melanomacrophages only, or a ‘bland proliferation of melanocytes’. Any tissue sampling technique is vulnerable to sampling a portion of the lesion which is not indicative of the most severe underlying pathology [1, 18]; TSEB is not unique in this regard. The authors do not believe that TSEB is more vulnerable to this limitation than other biopsy techniques, but further study with larger cohorts is warranted.

All three of the ‘diagnostically unsuccessful’ TSEBs were of lesions located anterior to the equator. Patient number 2 (see Table 2) underwent a second biopsy using a trans-scleral incisional biopsy (PLSU) approach, which confirmed choroidal melanoma. In some cases, incisional PLSU may be superior to Essen forceps when sampling anterior lesions; however, TSEB confers some distinct advantages over this technique. PLSU creates a larger wound with a deep scleral defect, increasing the risks of tumour seeding and compromising the integrity of the scleral coat [12, 18, 19]. PLSU is poorly suited to small, posterior lesions with challenging access. In our illustrative case report, for example, the location of the tumour would have made surgical incisional biopsy difficult and would have risked non-diagnostic biopsy or retinal penetration due to the shallow tumour.

In our series, we reported one case of retinal perforation with vitreous loss. The patient did not develop retinal detachment nor required further intervention for this complication. Nonetheless, TSEB confers several advantages over trans-vitreal biopsy. Firstly, with anterior choroidal and ciliary body tumours, it avoids the risk of lens touch. Secondly, it avoids vitrectomy and the associated risks, such as vitreous and subretinal haemorrhage, secondary retinal detachment and an increased rate of cataract formation [20]. Thirdly, the approach via pars plana ports means that the instrument can only be used at a single angle; therefore, for thin tumours, it may be difficult to gain sufficient samples without creating a large retinal defect. The Essen forceps can be used parallel or tangential to the inner scleral surface if utilised via the trans-scleral approach, which is particularly useful for shallow tumours. It is worth noting at this point that some centres now perform minimalist transretinal biopsy with the vitrectomy cutter, without vitrectomy, retinopexy or tamponade [10, 17, 21]. Unfortunately, this was not available at our services during the study period. Lastly, the technique yields much more tissue with more intact architecture. This allows easier histopathological interpretation of the pathology and permits numerous ancillary diagnostic tests such as immunohistochemistry and molecular pathology for diagnosis conformation and prognostication.

This study is limited by its retrospective nature and small sample size. Other factors, such as the experience of the surgeon, the available instrumentation and cost have not been examined. Further study with a larger cohort is warranted to continue to refine the indications and technique of TESB.

## Conclusion

Diagnosing posterior uveal tumours can be challenging in atypical cases. Essen forceps biopsy using a trans-scleral approach can enhance tissue sample quality and size in selected lesions, therefore increasing diagnostic accuracy with minimal risk. We recommend considering trans-scleral Essen forceps biopsy within the armamentarium of the ocular oncologist.

## Summary

### What was known before

- Identifying uveal melanoma may require intra-ocular biopsy in atypical cases.
- Established techniques include fine needle aspiration biopsy (FNAB) and trans-vitreal biopsy using vitreous cutters and Essen Forceps.

### What this study adds

- The trans-scleral Essen Forceps biopsy (TSEB) technique can improve diagnostic yield in atypical cases of uveal melanoma without compromising safety, even in shallow posterior lesions.

## Supplementary information

Eye reporting checklist

## Data Availability

The data that support the findings of this study are available from Moorfields Eye Hospital and The Royal Hallamshire Hospital, Sheffield but restrictions apply to the availability of these data, which were used under license for the current study, and so are not publicly available.
